# Comparison of *Rps* loci toward isolates, singly and combined inocula, of *Phytophthora sojae* in soybean PI 407985, PI 408029, PI 408097, and PI424477

**DOI:** 10.3389/fpls.2024.1394676

**Published:** 2024-07-01

**Authors:** Elizabeth M. Clevinger, Ruslan Biyashev, Clarice Schmidt, Qijian Song, Amine Batnini, Carlos Bolaños-Carriel, Alison E. Robertson, Anne E. Dorrance, M. A. Saghai Maroof

**Affiliations:** ^1^ School of Plant and Environmental Sciences, Virginia Tech, Blacksburg, VA, United States; ^2^ Department of Plant Pathology, Entomology and Microbiology, Iowa State University, Ames, IA, United States; ^3^ Soybean Genomics and Improvement Laboratory, Agricultural Research Service, Department of Agriculture, Beltsville, MD, United States; ^4^ Department of Plant Pathology, The Ohio State University, Wooster, OH, United States

**Keywords:** *Phytophthora sojae*, *Rps* genes, *Glycine max*, soybean, resistance breeding, recombinant inbred line population

## Abstract

For soybean, novel single dominant *Resistance to Phytophthora sojae* (*Rps)* genes are sought to manage Phytophthora root and stem rot. In this study, resistance to *P. sojae* was mapped individually in four recombinant inbred line (RIL) populations derived from crosses of the susceptible cultivar Williams with PI 407985, PI 408029, PI 408097, and PI424477 previously identified as putative novel sources of disease resistance. Each population was screened for resistance with five to seven isolates of *P. sojae* separately over multiple F_7_–F_10_ generations. Additionally, three of the populations were screened with inoculum from the combination of three *P. sojae* isolates (PPR), which comprised virulence to 14 *Rps* genes. Over 2,300 single-nucleotide polymorphism markers were used to construct genetic maps in each population to identify chromosomal regions associated with resistance to *P. sojae*. Resistance segregated as one or two genes to the individual isolates and one gene toward PPR in each population and mapped to chromosomes 3, 13, or 18 in one or more of the four RIL populations. Resistance to five isolates mapped to the same chromosome 3 region are as follows: OH7 (PI 424477 and PI408029), OH12168, OH7/8, PPR (PI 407985), and 1.S.1.1 (PI408029). The resistance regions on chromosome 13 also overlapped for OH1, OH25, OH-MIA (PI424477), PPR (PI 424477, PI 407985, and PI 408097), PPR and OH0217 (PI 408097), and OH4 (PI 408029), but were distinct for each population suggesting multiple genes confer resistance. Two regions were identified on chromosome 18 but all appear to map to known loci; notably, resistance to the combined inoculum (PPR) did not map at this locus. However, there are putative new alleles in three of four populations, three on chromosome 3 and two on chromosome 13 based on mapping location but also known virulence in the isolate used. This characterization of all the *Rps* genes segregating in these populations to these isolates will be informative for breeding, but the combined inoculum was able to map a novel loci. Furthermore, within each of these *P. sojae* isolates, there was virulence to more than the described *Rps* genes, and the effectiveness of the novel genes requires testing in larger populations.

## 1 Introduction

Soybean [*Glycine max* (L.) Merr.] is a major oil and protein crop that is grown worldwide. Soybean seed is composed mainly of 40% protein and 20% oil (Clemente and Cahoon, 2009). In the United States, the crop was planted on 87.5 million acres during the 2022 growing season and was worth $59.2 billion dollars in the 2021/2022 marketing year (http://www.soystats.com). *Phytophthora sojae* (Kaufmann and Gerdemann) is an economically important soybean pathogen in the United States and worldwide. Between 2015 and 2019, in 29 US states and Ontario, Canada, the estimated yield loss from *P. sojae* ranged from 25 to 29 million bushels annually (Bradley et al., 2021). The severity of loss depends on a wide variety of factors including soil type, compaction, and rainfall (Schmitthenner, 2000). *P. sojae* is a diverse organism with over 200 pathotypes (Dorrance, 2018a; Matthiesen et al., 2021; Hebb et al., 2022; McCoy et al., 2023). This pathogen causes root and stem rot in soybean plants, which can lead to stand and yield reduction in susceptible cultivars. Symptoms of *Phytophthora* root and stem rot (PRSR) early in the growing season include seed and seedling disease and pre- and post-emergence damping off. Later in the growing season, root rot and, in highly susceptible cultivars, stem lesions may develop if environmental and field conditions are favorable with *P. sojae* inoculum present (Schmitthenner, 1985; Dorrance et al., 2003a; Grau et al., 2004). Practices, such as crop rotation and tillage, are not as useful with this pathogen because oospores are able to persist in the soil and plant debris for years (Schmitthenner, 1985).

The two main types of resistance in soybean cultivars to *P. sojae* are single dominant resistance genes (*Rps* genes) and partial resistance. Partial resistance is quantitatively inherited, and several genes contribute to the level of resistance, whereas *Rps* genes are qualitatively inherited and are pathotype (formally race) specific (Schmitthenner, 1985; Grau et al., 2004; Dorrance, 2018b). The first *Rps* gene was identified in the 1950s (Bernard et al., 1957), and over 40 *Rps* genes and alleles have been mapped to date on 9 of the 20 soybean chromosomes (Dorrance, 2018a; Lin et al., 2022). In a genome-wide association study (GWAS), Van et al. (2020) identified 75 novel *Rps* loci for *P. sojae* on all chromosomes except 3 and 13 using 16 soybean panels, which included over 1,800 soybean accessions. However, only genes from three loci (*Rps1*, *Rps3*, and *Rps6*) have been used commercially, and these are *Rps1a*, *Rps1b*, *Rps1c*, *Rps1k*, *Rps3a*, and *Rps6* (Grau et al., 2004; Slaminko et al., 2010). The use of *Rps* genes has allowed for shifts in pathotype diversity of *P. sojae* toward those that are adapted to specific *Rps* genes. In the north central region of the United States, complex pathotypes of *P. sojae* are now pervasive (Dorrance et al., 2003b, 2016; Yan and Nelson, 2019; Matthiesen et al., 2021; Hebb et al., 2022; McCoy et al., 2022). In a pathotype diversity study of *P. sojae* in 11 states in the United States, over 200 unique pathotypes were observed many of which could cause disease on soybean with any of the commercially available *Rps* genes (Dorrance et al., 2016). Depending on the soybean-growing region, certain *Rps* genes were not as effective as in the past (Matthiesen et al., 2021; McCoy et al., 2022), and this highlights the need for continuing to identify and combine effective *Rps* genes during the development of new soybean cultivars or ensuring cultivars also have high partial resistance. Dorrance and Schmitthenner (2000) identified multiple sources of potentially novel *Rps* and quantitative resistance to PRSR in soybean accessions. The majority of the lines that showed resistance were from South Korea and included PI 407985, PI 408097, PI 408029, and PI 424477. These same PIs were also resistant toward *P. sojae* in a combined inoculum approach (Matthiesen et al., 2016) making them a high priority source for new *Rps* genes. South Korean accessions are genetically distant from US genotypes (Burnham et al., 2002), which makes them good candidates to expand the gene pool available to US breeding programs.

Of the four PIs, Gordon et al. (2007a) evaluated PI 408097 in F_2:3_ and F_2:4_ recombinant inbred line (RIL) populations and proposed that this PI may have a combination of *Rps* genes of *Rps1c* plus one of the following *Rps2*, *3a*, *3b*, or *4*. The objective of this study was to identify and map the potentially novel *Rps* loci in each of the RIL populations derived from crosses of Williams with PI 407985, PI 408029, PI 408097, and PI424477 using multiple generations. In this study, we used data from inoculations with single isolates of *P. sojae* and also three isolates of *P. sojae* that were combined before inoculation. This study allowed us to compare the results of these two different inoculation methods, construct high-density genetic maps, and determine chromosomal locations associated with resistance to PRSR using the four RIL populations. We utilized both single marker and quantitative approaches in mapping these loci. As qualitative and quantitative trait loci both have the same abbreviation (QTL), we have used Mendelian mapping as the term to indicate qualitative trait mapping. The findings from this study will expand the currently available *Rps* genes and genetics available for breeders to develop resistant cultivars.

## 2 Materials and methods

### 2.1 Plant material

Genetic material for this study included RIL populations PI 407985 × Williams, PI 408029 × Williams, PI 408097 × Williams, and PI 424477 × Williams, with 192, 188, 312, and 163 RILs, respectively. These RIL populations were created at Virginia Tech, Kentland Farm in Blacksburg, VA. The Plant Introduction (PI) lines: PI 407985, PI 408029, PI 408097, and PI 424477 were chosen based on results of previous *P. sojae* screens for resistance (Dorrance and Schmitthenner, 2000; Matthiesen et al., 2016). Williams is the differential with no *Rps* genes and is susceptible to all isolates of *P. sojae* (Dorrance et al., 2004). These four PI lines are from the US Soybean Germplasm Collection, and all are maturity group IV.

### 2.2 Rolled towel assay for phenotyping

Since PI 407985, PI 408029, PI 408097, and PI 424477 each contain several *Rps* genes, isolates of *P. sojae* were selected to potentially limit the expression to one or two *Rps* genes to enhance the possibility of fine mapping but also to identify one locus that would confer resistance to all the isolates. For each population, **Table 1** summarizes the number of RILs, the isolates used to inoculate, and the number of generations screened for disease reaction in a rolled towel assay (Dorrance et al., 2008).

**Table 1:** The *Phytophthora sojae* isolates and their associated pathotypes to determine segregation ratios for the three RIL populations, PI 407985 × Williams, PI 408097 × Williams, and PI 424477 × Williams, the number of RILs in each population, and the generation(s) following hypocotyl inoculation in rolled towel assays.

Population and number of RILs	*P. sojae* isolates and (pathotypes)^a^	Generation assayed
PI 407985 × Williams (192 RILs)	OH1 (vir 7)	F_7_, F_8_, F_9_
	OH25 (vir 1a, 1b, 1c, 1k, 5, 7)	F_7_, F_8_, F_9_
	OH7/8 (vir 1a, 2, 3a, 3c, 4, 5, 6, 7, 8)	F_8_
	OH12168 (vir 1a, 1b, 2, 3a, 3c, 4, 5, 6, 7, 8)	F_8_, F_9_
	OH-Windfall (vir 1a,1b, 1k, 2, 3a, 3b, 5, 7, 8)	F_8_, F_9_
	PPR (vir 1a, 1b, 1c, 1d, 1k, 2, 3a, 3b, 3c, 4, 5, 6, 7, 8)	F_9_
PI 408029 × Williams (188 RILs)	OH1 (vir 7)	F_9_
	OH4 (vir 1a, 1c, 7)	F_9_
	OH7 (vir 1a, 3a, 3c, 4, 5, 6, 7)	F_9_
	OH25 (vir 1a, 1b, 1c, 1k, 5, 7)	F_9_
	1.S.1.1 (vir 1a, 1b, 1k, 2, 3a, 3c, 4, 5, 6, 7, 8)	F_9_
PI 408097 × Williams (312 RILS)	OH1 (vir 7)	F_8_, F_9_, F_10_, F_11_
	OH2 (vir 1b, 5, 7)	F_8_, F_9_
	OH0217 (vir 1a, 1c, 1k, 3b, 6, 7)	F_8_
	OH7 (vir 1a, 3a, 3c, 4, 5, 6, 7)	F_9_
	OH-Dayton (vir 1a, 1b, 1c, 1k, 2, 3a, 5, 7)	F_8_
	OH-MIA (vir 1a, 1b, 1c, 2, 3a, 5, 7, 8)	F_8_, F_11_
	OH-Windfall (vir 1a, 1b, 1k, 2, 3a, 3b, 5, 7, 8)	F_9_, F_11_
	PPR (vir 1a, 1b, 1c, 1d, 1k, 2, 3a, 3b, 3c, 4, 5, 6, 7, 8)	F_10_
PI 424477 × Williams (163 RILs)	OH1 (vir 7)	F_7_
	OH7 (vir 1a, 3a, 3c, 4, 5, 6, 7)	F_7_
	OH25 (vir 1a, 1b, 1c, 1k, 5, 7)	F_7_
	OH-Dayton (vir 1a, 1b, 1c, 1k, 2, 3a, 5, 7)	F_7_
	OH-MIA (vir 1a, 1b, 1c, 2, 3a, 5, 7, 8)	F_7_
	PPR (vir 1a, 1b, 1c, 1d, 1k, 2, 3a, 3b, 3c, 4, 5, 6, 7, 8)	F_9_

a The pathotypes of each OH isolate were determined on the following differentials: Williams (rps), Harlon (Rps1a), Harosoy 13XX (Rps1b), Williams 79 (Rps1c); PI 103091 (Rps1d); Williams82 (Rps1k); L76–1988 (Rps2); L83–570 (Rps3a); PRX 146–36 (Rps3b); PRX 145–48 (Rps3c); L85–2352 (Rps4); L85–3059 (Rps5); Harosoy 62XX (Rps6), Harosoy (Rps7), and PI 399073 (Rps8). PPR is the combined isolates of PT2004 C2.S1 (pathotypes 1a, 1b, 1c, 1d, 1k, 2, 3c, 4, 6, 7), P7074 (pathotypes 1b, 1d, 2, 3a, 3b, 3c, 4, 5, 6, 7), and R26 (pathotypes 1b, 1d, 2, 3a, 3b, 4, 5, 6, 7, 8).

#### 2.2.1 Screening with single *P. sojae* isolates

At Ohio State University (OSU), single isolates of *P. sojae* were used to map as many of the loci present in these PI lines as possible. In Ohio, *P*. *sojae* isolate OH1 was selected as it has virulence to only one known *Rps* gene, *Rps*7, and could identify the RILs that were lacking in *Rps* genes. This served as a check for subsequent inoculations to ensure the same lines were susceptible to all remaining isolates. Initially, isolates were transferred to Petri dishes with lima bean (*Phaseolus lunatus* L.) agar media (LBA) (lima beans 50 g/l, agar 12 g/l) and placed in a 25°C incubator. Isolates (virulence pathotype) OH1 (vir 7), OH2 (vir 1b, 7), OH4 (vir 1a, 1c, 7), OH7 (vir 1a, 3a, 3c, 4, 5, 6, 7), OH7/8 (vir 1a, 2, 3a, 3c, 4, 5, 6, 7, 8), OH12168 (vir 1a, 1b, 2, 3a, 3c, 4, 5, 6, 7, 8), OH0217 (vir 1a, 1c, 1k, 3b, 6, 7), OH25 (vir 1a, 1b, 1c, 1k, 7), OH-Dayton (vir 1a, 1b, 1c, 1k, 2, 3a, 5, 7), OH-MIA (vir 1a, 1b, 1c, 2, 3a, 5, 7, 8), 1.S.1.1 (vir 1a, 1b, 1k, 2, 3a, 3c, 4, 5, 6, 7, 8), and OH-Windfall (vir 1a, 1b, 1k, 2, 3a, 3b, 5, 7, 8) were used to inoculate the hypocotyls of 7-day-old seedlings in a rolled towel assay (10 to 15 seedlings/RIL) (Dorrance et al., 2008). After 7 days, germination towels were unrolled, and seed coats, twisted seedlings, and non-germinated seeds were removed. Petri dishes were fully colonized with 7-to-8-day old individual *P. sojae*. The colony was cut and placed in a syringe and forced through an 18-gauge hypodermic syringe to make a slurry. A small incision was made approximately 1 cm below the cotyledon on the surface of the hypocotyl on each seedling. The mycelial slurry (LBA and *P. sojae* mycelia) covered the wound, and the rolled towels were reassembled and incubated at 25°C and 50% HR in a growth chamber. Differential checks each having one known *Rps* gene or none included in each assay were Harlon (*Rps1a*), Harosoy 13XX (*Rps1b*), Williams 79 (*Rps1c*); PI 103091 (*Rps1d*); Williams82 (*Rps1k*); L76–1988 (*Rps2*); L83–570 (*Rps3a*); PRX 146–36 (*Rps3b*); PRX 145–48 (*Rps3c*); L85–2352 (*Rps4*); L85–3059 (*Rps5*); and Harosoy 62XX (*Rps6*), Harosoy (*Rps7*), PI 399073 (*Rps8*), and Williams (*rps*). Seven days after inoculation, seedlings were scored for presence of lesions. First, the response of the differential checks was examined to determine if the isolate had the expected virulence to the each known *Rps* gene. If the response was as expected, the RILs in the population were scored. The trait is a single dominant gene, and as such, a categorical score for the RILs was used based on the number of seedlings that developed expanding lesions from the total inoculated. For each isolate, where resistant (R), segregating (H), or susceptible (S), for 0% up to 20%, 21% to 79%, or 80% to 100%, respectively, scores were given. Due to the nature of the assay, inoculating the hypocotyl with a hypodermic, there are successful and unsuccessful infections, which is why the categories are broad. Due to the limited seed, the phenotype assay was done only once for isolates where a clear segregation pattern was observed and the differentials’ response as expected. Comparisons were made across isolates to ensure those RILs identified as susceptible to OH1 were consistent across all isolates. Furthermore, only data where differentials responded as expected were used.

#### 2.2.2 Screening with a combination of three *P. sojae* isolates

At Iowa State University (ISU), isolates with all known virulence to *Rps* loci were combined prior to inoculation to identify the unique loci as previously described (Matthiesen et al., 2016). Briefly, three *P. sojae* isolates, PT2004 C2.S1 (pathotype 1a, 1b, 1c, 1d, 1k, 2, 3c, 4, 6, 7), P7074 (pathotype 1b, 1d, 2, 3a, 3b, 3c, 4, 5, 6, 7), and R26 (pathotype 1b, 1d, 2, 3a, 3b, 4, 5, 6, 7, 8), were grown on separate plates containing dilute DV8 media (40 ml of V-8 juice, 0.6 g of CaCO3, 0.2 g of Bacto yeast extract, 1 g of sucrose, 0.01 g of cholesterol, 20 g of Bacto agar, 1 L of distilled water) amended with neomycin sulfate (50 µg/ml) and chloramphenicol (10 µg/ml) for 7 to 8 days at 25°C in the dark. Each isolate slurry was prepared by combining each isolate grown separately in a 1:1:1 mixture used for the hypocotyl inoculation described above for each soybean line. The combined three isolates of PT2004 C2.S1, P7074, and R26 will be referred to as PPR, hereafter. Rolled towels with inoculated seedlings were kept at 25°C with a 16-h photoperiod for 7 days before percentage of killed seedlings for each RIL was scored. The pathogenicity of the 1:1:1 mixture was checked on the following 14 differentials: L88–8470 (*Rps1a*), L77–1863 (*Rps1b*), Williams 79 (*Rps1c*), L93–3312 (*Rps1d*), Williams 82 (*Rps1k*), L82–1449 (*Rps2*), L83–570 (*Rps3a*), L91–8347 (*Rps3b*), L92–7857 (*Rps3c*), L85–2352 (*Rps4*), L85–3059 (*Rps5*), L89–1581 (*Rps6*), L93–3258 (*Rps7*), PI 399073 (*Rps8*), and Sloan (*rps*). RIL responses were scored susceptible if at least 70% of seedlings died on all differentials checks. The experiment was replicated two times. The inoculated RILs were scored homozygous resistant if <30% and susceptible if more than 70% of the RILs developed symptoms. All others were classified as heterozygous.

### 2.3 Molecular marker assay

For single-nucleotide polymorphism (SNP) and simple sequence repeat (SSR) marker genotyping, unfolded first or second trifoliate leaves of greenhouse-grown plants were collected for DNA extraction. DNA from parental lines and at least 10 bulked plants from each individual RIL was isolated from lyophilized tissues using the CTAB method as described by Saghai-Maroof et al. (1984) with minor modifications. DNA concentration was measured with a Qubit 3.0 Fluorometer (Life Technologies, Carlsbad, CA).

To identify chromosomal regions associated with resistance to *P. sojae*, the four RIL populations and parents were genotyped using the Illumina Infinium BARCSoySNP6K BeadChip (Song et al., 2020) at USDA-ARS, Soybean Genomics and Improvement Lab, Beltsville, MD. The marker data sets were processed using GenomeStudio software (version 3.2.23). SNP markers that were monomorphic between parents of each RIL population and those that had more than 20% missing data were not used for linkage map construction. SSR markers were added to two critical *P. sojae* regions on chromosomes 3 and 18 in PI 407985 × Williams and PI 408097 × Williams. SSR markers were amplified by PCR with dye-labeled forward primers (Song et al., 2010) and analyzed by capillary electrophoresis using an Applied Biosystems 3130xl Genetic Analyzer (Foster City, CA).

### 2.4 Map construction, Mendelian and quantitative trait locus analysis

The genetic maps were constructed using JoinMap 4.0 (van Ooijen, 2006) based on an LOD threshold of 4.0 and a maximum recombination frequency of 50% for the original grouping. Marker order and their positions within each linkage group were determined using the maximum likelihood algorithm and Kosambi mapping function; those unassigned to any linkage group were excluded. Mendelian inheritance of the resistant trait was mapped with each isolate individually due to the number of different RILs used in each inoculation and generation.

MapQTL 5 software (van Ooijen, 2004) was used for the identification of each quantitative trait locus for reaction to each of the *P. sojae* isolates assayed in this study. Scores of individual RILs were used on the scale of 1 = homozygous resistant, 2 = heterozygous, and 3 = homozygous susceptible. The QTL analysis was performed in two stages: interval mapping (IM) to reveal critical chromosomal regions followed by more detailed QTL mapping provided by the enhanced power of composite interval mapping (CIM) with the walking speed set to 1 cM.

To identify levels of LOD significance thresholds on a genome-wide basis, 1,000 iteration permutation tests were conducted. Calculated genome-wide LOD thresholds were used as a base line in significant QTL justification. Surprisingly, some minor QTLs were identified, which is unexpected for this type of resistance. Our results focused on only the major QTL and those loci that could be mapped with both Mendelian and QTL mapping.

## 3 Results

### 3.1 Phenotypic data of RILs

Based on Chi-square analysis of the phenotypic data following inoculation, resistance toward one or more of the isolates alone or to the combined inoculum, PPR, in each of the RIL populations segregated as one to two *Rps* genes (**Tables 2**, **3**). *P. sojae* isolate OH1 has virulence to one known *Rps* gene, *Rps7*, and resistance to OH1 segregated as two genes in each of these populations. This indicates that the total number of genes we expected to map in each of the populations was two. For each of these advanced RIL populations, segregation to the remaining isolates did not follow normal phenotypes for single or two-gene inheritance, with a maintenance of heterozygotes across the generations. This has been noted in other studies; thus, the chi-square analysis was based on the combination of RILs classified as heterozygous with either the homozygous-resistant or homozygous-susceptible RILs. Resistance was conferred by one *Rps* gene toward *P. sojae* OH12168, OH7/8, and the combined inoculum (PPR) in the PI 407985 × Williams based on segregation ratios. In addition, in this population, resistance toward *P. sojae* OH25 and OH-Windfall was conferred by one or two *Rps* genes. Resistance in the PI 408029 × Williams toward *P. sojae* isolates OH7 and 1.S.1.1 was conferred by one *Rps* gene, while for OH4 and OH25, resistance was conferred by either one or two *Rps* genes. In the PI 408097 × Williams population, resistance toward *P. sojae* isolates OH2, OH-Dayton, and PPR was controlled by two *Rps* genes, while resistance toward *P. sojae* isolates OH-MIA, OH0217, and OH-Windfall may be conferred by one or two *Rps* genes. In the PI 424477 × Williams population, resistance toward *P. sojae* isolates OH7, OH-MIA, OH25, and PPR were controlled by a single *Rps* gene.

**Table 2:** Chi-square table for PI 407985 × Williams RIL and PI 408029 × Williams populations across generations following inoculation with five isolates of *Phytophthora sojae* separately and inoculation with inoculum of three isolates combined (PPR).

*P. sojae* isolate	Generation	Number of RILs	Phenotypic grouping^a^	No. Rps genes	R or R+H	S or S+H	No. of H	Chi-square	Significance
PI 407985 × Williams									
OH1*	F_9_	186	R:H+S	2	134	52	27	0.80	ns
OH1	F_9_	181	R+H:S	2	142	39	24	1.29	ns
OH25	F_7_	154	R:H+S	1	74	80	45	0.15	ns
OH25	F_7_	154	R+H:S	2	119	35	45	0.46	ns
OH25	F_8_	161	R:H+S	1	84	77	24	0.37	ns
OH25	F_9_	186	R+H:S	2	137	49	27	0.17	ns
OH25*	F_9_	186	R:H+S	1	100	86	35	1.16	ns
OH25*	F_9_	186	R+H:S	2	135	51	35	0.54	ns
OH25*	F_9_	185	R+H:S	1	97	88	63	0.41	ns
OH12168	F_8_	162	R:H+S	1	70	92	15	2.67	ns
OH12168	F_8_	162	R+H:S	1	85	77	15	0.31	ns
OH12168*	F_9_	186	R:H+S	1	89	97	24	0.31	ns
OH12168*	F_9_	184	R+H:S	1	97	87	22	0.50	ns
OH-Windfall	F_8_	162	R:H+S	1	88	74	39	1.39	ns
OH-Windfall	F_8_	162	R+H:S	2	127	35	39	1.11	ns
OH7/8	F_8_	162	R+H:S	1	81	81	15	0.01	ns
PPR^+*^	F_9_	186	R+H:S	1	91	95	36	0.10	ns
PI 408029 × Williams									
OH1	F_9_	154	R:H+S	2	113	41	18	0.22	ns
OH4	F_9_	127	R:H+S	1	67	60	30	0.40	ns
OH4	F_9_	127	R+H:S	2	97	30	30	0.13	ns
OH7	F_9_	136	R:H+S	1	65	71	24	0.25	ns
OH25	F_9_	167	R+H:S	2	128	39	33	0.23	ns
1.S.1.1	F_9_	111	R:H+S	1	52	59	13	0.43	ns

*Indicates where the results from two replications of an isolate were combined into a single dataset. All the other isolates represent one replication.

^+^PPR is the combined isolates of PT2004 C2.S1 (pathotypes 1a, 1b, 1c, 1d, 1k, 2, 3c, 4, 6, 7), P7074 (pathotypes 1b, 1d, 2, 3a, 3b, 3c, 4, 5, 6, 7), and R26 (pathotypes 1b, 1d, 2, 3a, 3b, 4, 5, 6, 7, 8).

a R indicates homozygous resistant, H indicates heterozygous, and S indicates homozygous susceptible based on the number of seedlings in each RIL that were resistant (0% to 20%); heterozygous (21%–79%), and susceptible (80%–100%) following inoculation in a rolled towel assay.

**Table 3:** Chi-square table for PI 408097 × Williams and for PI 424477 **×** Williams RIL populations across generations following inoculation with six and four isolates of *Phytophthora sojae* and inoculation with inoculum of three isolates combined (PPR).

*P. sojae* isolate	Generation	Number of RILs	Phenotypic grouping^a^	No. *Rps* genes	R or R+H	S or S+H	No. of H	Chi-square	Significance
PI 408097 × Williams									
OH1	F_9_	254	R:H+S	2	182	72	23	1.52	Ns
OH2	F_9_	261	R:H+S	2	191	70	35	0.46	Ns
OH-MIA*	F_8_	263	R:H+S	1	122	141	76	1.24	Ns
OH-MIA*	F_8_	263	R+H:S	2	198	65	76	0.01	Ns
OH0217	F_8_	242	R:H+S	1	128	114	57	0.93	Ns
OH0217	F_8_	242	R+H:S	2	185	57	57	0.27	Ns
OH-Dayton	F_8_	259	R+H:S	2	197	62	85	0.16	Ns
OH-Windfall	F_10_	267	R:H+S	1	126	141	78	0.58	Ns
OH-Windfall	F_10_	267	R+H:S	2	204	63	78	0.28	Ns
PPR^+*^	F_10_	256	R:H+S	2	199	57	12	1.02	Ns
PI 424477 × Williams									
OH1	F_7_	149	R+H:S	2	113	36	21	0.06	Ns
OH25*	F_7_	154	R+H:S	1	88	77	77	1.40	Ns
OH7	F7	148	R+H:S	1	71	77	21	0.35	Ns
OH-MIA	F7	149	R+H:S	1	83	66	45	1.68	Ns
PPR^+^*	F_9_	156	R+H:S	1	73	83	17	0.68	Ns

*Indicates where the final results from two replications of an isolate were combined into a single dataset. The remaining phenotypic data are from one replication.

^+^PPR is the combined isolates of PT2004 C2.S1 (pathotypes 1a, 1b, 1c, 1d, 1k, 2, 3c, 4, 6, 7), P7074 (pathotypes 1b, 1d, 2, 3a, 3b, 3c, 4, 5, 6, 7), and R26 (pathotypes 1b, 1d, 2, 3a, 3b, 4, 5, 6, 7, 8).

a R indicates homozygous resistant, H indicates heterozygous, and S indicates homozygous susceptible based on the number of seedlings in each RIL that were resistant (0% to 20%), heterozygous (21%–79%), and susceptible (80%–100%) following inoculation in a rolled towel assay.

### 3.2 Mendelian inheritance (qualitative trait loci) and quantitative trait loci for single *Rps* gene mapping

Prior to mapping the resistance phenotype, the accuracy of the constructed genetic map for each of the four RIL populations was verified against the soybean genome sequence assembly version Wm82.a2 (http://soybase.org, Brown et al., 2021). The total number of mapped markers was 2,316 for the population PI 407985 × Williams, 2,670 for PI 408029 × Williams, 2,644 for PI 408097 × Williams, and 2,477 for PI 424477 × Williams. The high-density genetic maps were subsequently used to identify chromosomal regions associated with resistance to *P. sojae* isolates in these populations.

**Figure 1:**
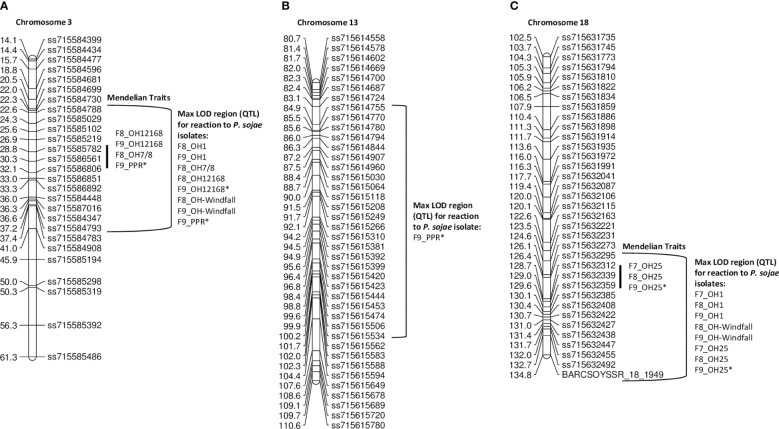
**(A)** Chromosome 3 regional map for Mendelian and QTL mapping in PI 407985 **×** Williams (positions of *Rps* loci in the inner bracket denoted by a black line). The max LOD region for reaction to isolates OH1, OH7/8, OH12168, OH-Windfall, and PPR are denoted by the bracketed region to the right side of the map. **(B)** Chromosome 13 regional map for QTL mapping in the PI 407985 × Williams. The bracketed region is the max LOD region for reaction to the combined inoculum, PPR. **(C)** Chromosome 18 regional map for Mendelian and QTL mapping in PI 407985 **×** Williams (positions of *Rps* loci in the inner bracket denoted by a black line). The max LOD region for the reaction to isolates OH1, OH25, and OH-Windfall are denoted by the bracketed region to the right side of the map. Isolates designated with an asterisk (*) indicate that disease screening results from two replications were combined into a single dataset; the remaining are from one replication. All isolates were mapped separately and then placed on the map.

For the **PI 407985 × Williams** population, 192 RILs were phenotyped over three generations, F_7_, F_8_, and F_9_ (**Table 4**; **Supplementary Table 1**). Resistance toward OH7/8, OH12168, and combined inoculum (PPR) all segregated as a single gene and mapped to the same region on chromosome 3, as well as resistance toward the remaining isolates OH1 and OH-Windfall, which were confirmed via QTL mapping (**Figure 1A**, **Supplementary Figure 1**; **Table 4**). The SNP marker, ss715585782, was at the maximum LOD for OH1, OH7/8, OH12168, and PPR consistently across the F_8_ and F_9_ generations (**Supplementary Figure 1A**) indicating that the same *Rps* gene may be conferring resistance and could be a new allele based on the virulence pathotypes. The maximum LOD for OH-Windfall mapped ~3.6 cM below ss715585782 near the marker ss715586806 (**Table 4**). This region covers 1.31 Mb and is enriched for genes encoding leucine-rich repeat containing proteins as well as serine threonine kinase based on the Wm82.a2 sequence assembly (www.soybase.org, Brown et al., 2021) (**Supplementary Table 2**).

**Table 4:** Resistance in PI 407985 **×** Williams toward *Phytophthora sojae* in F_7_, F_8_, and F_9_ of the RIL population based on QTL mapping.

Gen. (year assayed)	*P. sojae* isolate	QTL on chr.	LOD	% explained variation	Marker at Max LOD	Flanking markers
F_8_ (2017)	OH1	3	8.12	21.2%	ss715585782	ss715584788 ss715584793
F_9_ (2018)	OH1*	3	13.98	29.9%	ss715585782	
F_9_ (2023)	OH1	3	22.0	43.5%	ss715585782	
F_8_ (2017)	OH7/8	3	100	100%	ss715585782	
F_8_ (2017)	OH12168	3	100	100%	ss715585782	
F_9_ (2018)	OH12168*	3	78.89	88.4%	ss715585782	
F_9_ (2023)	OH12168*	3	76.61	88.8%	ss715585782	
F_8_ (2017)	OH-Windfall	3	5.51	14.6%	ss715586806	
F_9_ (2019)	OH-Windfall	3	4.46	10.5%	ss715586806	
F_9_ (2022)	PPR*^+^	3	41.87	73.1%	ss715585782	
F_9_ (2022)	PPR*^+^	13	5.82	16.1%	ss715615453	ss715614755 ss715615534
F_7_ (2016)	OH1	18	6.64	18.5%	ss715632359	ss715632295 BARCSoySSR_18_1949
F_8_ (2017)	OH1	18	13.79	32.9%	ss715632359	
F_9_ (2018)	OH1*	18	11.26	24.4%	ss715632312	
F_9_ (2023)	OH1	18	6.94	16.4%	ss715632492	
F_7_ (2016)	OH25	18	31.44	62.4%	ss715632312	
F_8_ (2017)	OH25	18	30.98	59.3%	ss715632312	
F_9_ (2018)	OH25*	18	57.72	76.9%	ss715632312	
F_9_ (2019)	OH25	18	33.63	56.8%	ss715632312	
F_9_ (2023)	OH25*	18	42.94	66.3%	ss715632312	
F_8_ (2017)	OH-Windfall	18	23.34	48.8%	ss715632359	
F_9_ (2019)	OH-Windfall	18	28.23	50.8%	ss715632359	

*Indicates where the final results are from two replications of an isolate combined into a single dataset. The remaining are from one replication.

^+^PPR is the combined isolates of PT2004 C2.S1 (pathotypes 1a, 1b, 1c, 1d, 1k, 2, 3c, 4, 6, 7), P7074 (pathotypes 1b, 1d, 2, 3a, 3b, 3c, 4, 5, 6, 7), and R26 (pathotypes 1b, 1d, 2, 3a, 3b, 4, 5, 6, 7, 8).

Interestingly, resistance toward the combined inoculum, PPR, segregated as a single gene in the F_9_ generation, but a minor QTL on chromosome 13 explaining 16.1% of the phenotypic variation with an LOD of 5.82 was identified (**Table 4**). This region was flanked by SNP markers, ss715614755 and ss715615534 (**Figure 1B**). There are 28 genes that encode for leucine-rich repeats in this large region (5.4 Mb) based on the Wm82.a2 sequence (soybase.org).

Resistance to *P. sojae* isolates OH1, OH25, and OH-Windfall mapped on chromosome 18. The flanking markers were the same across the F_7_, F_8_, and F_9_ generations (**Table 4**, **Figure 1C**; **Supplementary Figure 1B**). Based on Mendelian mapping, resistance toward OH-Windfall mapped 9 cM below the end marker BARCSOYSSR_18_1949 on chromosome 18, which was also a flanking marker based on QTL mapping. However, the marker at the peak LOD for this isolate was ss715632359, which maps near the other isolates (**Figure 1C**, bottom of **Table 4**). Within this physical region (271,334 bp), there are four annotated disease resistance genes (*Glyma.18g282100, Glyma.18g282600, Glyma.18g283200,* and *Glyma.18g284100*) based on Glyma82.a2.v1 (soybase.org). It is important to note that the RILs did not respond consistently to these isolates for resistance or susceptibility indicating the possibility that this region may have several *Rps* genes to *P. sojae* that confer resistance to these isolates. Additionally, any of the known *Rps* genes (*Rps4*, *5*, and *6*) on chromosome 18 would confer resistance to OH25. However, OH-Windfall has virulence to *Rps5*, thus *Rps4* and *Rps6* are likely candidates.

**Figure 2:**
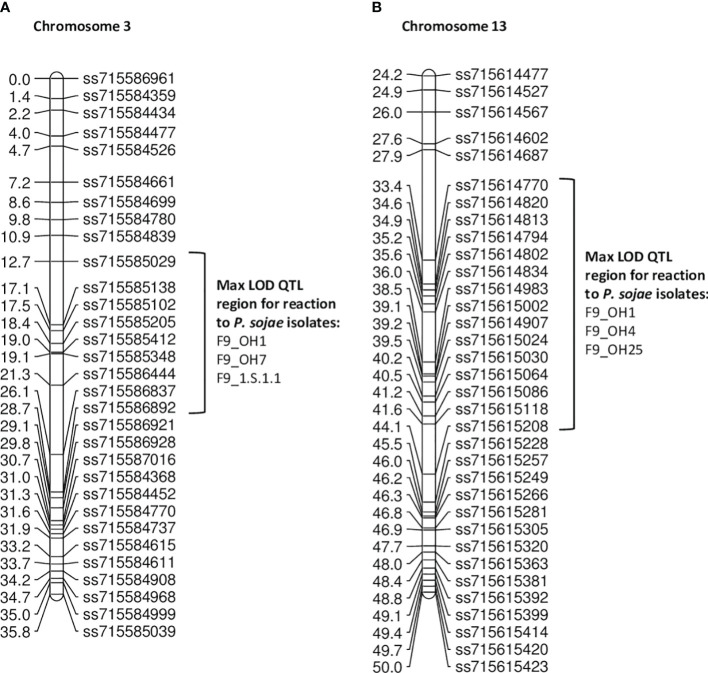
**(A)** Chromosome 3 regional map for QTL mapping in PI 408029 × Williams. The max LOD region for reaction to isolates OH1, OH7, and 1.S.1.1 are denoted by the bracketed region to the right side of the map. **(B)** Chromosome 13 regional map for QTL mapping in PI 408029 × Williams. The max LOD region for reaction to isolates OH1, OH4, and OH25 are denoted by the bracketed region to the right side of the map.

In the **PI 408029 × Williams** population, 188 RILs were screened in the F_9_ generation with five single isolates (**Table 5**). Resistance toward OH1 mapped to chromosomes 3 and 13 (**Figures 2A, B**). The marker at the maximum LOD score with QTL mapping was ss715585348 for the three isolates (OH1, OH7, 1.S.1.1) potentially indicating the same gene confers resistance (**Table 5**; **Supplementary Figure 2A**). This region from ss715585348 to ss715586444 covers less than 1 Mb at 805,038 bp and partially overlaps the *Rps* gene region identified in the PI 407985 × Williams RIL population, which contains sequences of many NBS-LRR genes. Based on the virulence to known *Rps* genes in these isolates (*Rps1a*, *Rps1b*, and *Rps1k*), there is the possibility that *Rps1c* could be in PI 408029 and contributing to resistance.

**Table 5:** Resistance in PI 408029 × Williams toward *Phytophthora sojae* in the F_9_ of the RIL population based on QTL mapping.

Gen. (year assayed)	*P. sojae* isolate	QTL on chr.	LOD	% explained variation	Marker at Max LOD	Flanking markers
F_9_ (2023)	OH1	3	9.59	25.8%	ss715585348	ss715585029 ss715586892
F_9_ (2023)	OH7	3	28.74	67.4%	ss715585348	
F_9_ (2023)	1.S.1.1	3	44.33	90.3%	ss715585348	
F_9_ (2023)	OH1	13	9.98	25.9%	ss715615002	ss715614770 ss715615208
F_9_ (2023)	OH4	13	18.16	49.1%	ss715615024	
F_9_ (2023)	OH25	13	14.05	32.4%	ss715615002	

Resistance towards *P. sojae* isolate OH4 mapped below that of OH1 and OH25 on chromosome 13 with SNP marker, ss715615024, at the maximum LOD (**Figure 2B**). The similarity of the three LOD curves for this chromosomal region emphasizes the consistency of the map location over all three isolates (**Supplementary Figure 2B**). Based on the Wm82.a2 assembled sequence, this region encompasses ~0.8 Mb, and there are 12 genes with annotations of leucine-rich repeat and defense related. These three isolates, OH1, OH4, and OH25, do not have any virulence to known *Rps* genes that map to this region, but this does not necessarily preclude that there are no additional alleles of these genes (*Rps*3a, 3b, 3c, and 8) present in this PI.

**Figure 3:**
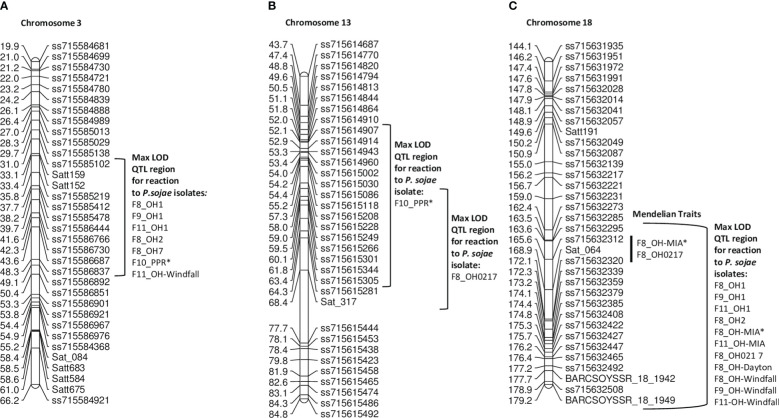
**(A)** Chromosome 3 regional map for Mendelian and QTL mapping in PI 408097 **×** Williams (positions of *Rps* loci in the inner bracket denoted by a black line). The max LOD region for reaction to isolates OH1, OH2, OH7, OH-Windfall, and PPR are denoted by the bracketed region to the right side of the map. **(B)** Chromosome 13 regional map for QTL mapping in PI 408097 **×** Williams. The max LOD region for reaction to isolates PPR and OH0217 are denoted by the bracketed regions to the right side of the map. **(C)** Chromosome 18 regional map for Mendelian and QTL mapping in PI 408097 **×** Williams (positions of *Rps* loci in the inner bracket denoted by a black line). The max LOD region for reaction to isolates OH1, OH2, OH0217, OH-Windfall, OH-MIA, and OH-Dayton are denoted by the bracketed region to the right side of the map. Isolates designated with an asterisk (*) indicate that disease screening results from two replications were combined into a single dataset; the remaining are from one replication.

The largest of the four RIL populations, **PI 408097 × Williams** 312 RILs were screened over four generations (F_8_, F_9_, F_10_, F_11_) with seven single isolates and combined inoculum (PPR). For this population, QTL mapping was primarily used as there were two genes segregating for resistance toward *P. sojae* isolates OH1, OH2, OH-Dayton, and PPR, while OH-MIA, OH0217, and OH-Windfall were difficult to discern between one and two *Rps* genes. As such, resistance was mapped to three regions on chromosomes 3, 13, and 18 (**Table 6**, **Figures 3A–C**; **Supplementary Figure 3A, B**) with QTL mapping only.

**Table 6:** Resistance in PI 408097 **×** Williams toward *Phytophthora sojae* in F_8_, F_9_, F_10_, and F_11_ of the RIL population based on QTL mapping.

Gen. (year assayed)	*P. sojae* isolate	QTL on chr.	LOD	% explained variation	Marker at Max LOD	Flanking markers
F_8_ (2016)	OH1	3	22.87	35.9%	ss715586444	ss715585102 ss715586837
F_9_ (2017)	OH1	3	20.36	30.9%	ss715586444	
F_11_ (2023)	OH1	3	18.72	29.2%	ss715586444	
F_8_ (2016)	OH2	3	23.85	36.7%	ss715586444	
F_8_ (2016)	OH7	3	44.55	59.6%	ss715586444	
F_11_ (2023)	OH-Windfall	3	12.09	19.1%	ss715585412	
F_10_ (2019)	PPR*^+^	3	21.07	27.4%	Satt159	
F_10_ (2019)	PPR*^+^	13	7.15	10.2%	ss715615002	ss715614907 ss715615305
F_8_ (2016)	OH0217	13	4.47	8.3%	ss715615118	ss715615086 Sat_317
F_8_ (2016)	OH1	18	4.60	8.3%	ss715632320	ss715632295 BARCSOYSSR_18_1949
F_9_ (2017)	OH1	18	7.26	12.0%	ss715632320	
F_11_ (2023)	OH1	18	6.09	10.6%	ss715632320	
F_8_ (2016)	OH2	18	4.86	8.7%	ss715632320	
F_8_ (2016)	OH-MIA*	18	50.62	59.9%	ss715632320	
F_11_ (2023)	OH-MIA	18	9.81	18.6%	ss715632320	
F_8_ (2016)	OH0217	18	37.36	51.6%	ss715632320	
F_8_ (2016)	OH-Dayton	18	35.62	47.4%	ss715632320	
F_8_ (2016)	OH-Windfall	18	18.93	29.1%	ss715632339	
F_9_ (2017)	OH-Windfall	18	13.29	20.9%	ss715632320	
F_11_ (2023)	OH-Windfall	18	11.22	17.8%	ss715632320	

*Indicates where the final results are from two replications of an isolate combined into a single dataset. The remaining are from one replication.

^+^PPR is the combined isolates of PT2004 C2.S1 (pathotypes 1a, 1b, 1c, 1d, 1k, 2, 3c, 4, 6, 7), P7074 (pathotypes 1b, 1d, 2, 3a, 3b, 3c, 4, 5, 6, 7), and R26 (pathotypes 1b, 1d, 2, 3a, 3b, 4, 5, 6, 7, 8).

The max LOD QTL region on chromosome 3 was flanked by SNP markers ss715585102 and ss715586837 for OH1, OH2, OH7, OH-Windfall, and PPR (**Figure 3A**, **Table 6**). However, the peak SNP marker differed for OH-Windfall and PPR from that of the others. In terms of physical distance, this interval covers 2.2 Mb. There are six NBS-LRR genes within a 240-kb region of the peak markers ss71586444, *Glyma.03g035300, Glyma.03g037000, Glyma.03g037100, Glyma.03g037300, Glyma.03g037400,* and *Glyma.03g037900*. The resistance genes, *Rps1a*, *Rps1b*, *Rps1c*, *Rps1d*, *Rps1k*, and *Rps7*, do not confer resistance to both of these isolates, thus potentially, resistance to PPR and OH-Windfall could be new *Rps* genes, as there were no NBS-LRR genes within the 240 kb of each of these markers in the Wm82.a2 sequence (soybase.org). Also noteworthy is that more than one location on chromosome 3 was mapped at this locus and could be contributing to the maintenance of heterozygosity in this population to these isolates.

Resistance was mapped toward isolates OH0217 and PPR on chromosome 13 near markers ss715615002 and ss715615118, respectively (**Figure 3B**). These two resistance loci were approximately 1 cM apart, and for both, the LOD was <7.15 and explained ¾10.2% of the genetic variation (**Table 6**). This is unexpectedly low for a dominant R-gene. This could be a novel gene or potentially incomplete expression of an R gene on chromosome 13 as *Rps3a*, *3b*, *3c*, and *8* are not effective against PPR, while *Rps3a*, *3c*, and *8* are effective toward OH0217. This region of 2 Mb has 26 genes annotated with LRR domains.

Resistance to OH1, OH2, OH-MIA, OH0217, OH-Dayton, and OH-Windfall all mapped to chromosome 18 (**Figures 3C**). As mentioned above, resistance was segregating as two genes toward these isolates; thus, the second gene was detected in the mapping process. The marker at the maximum LOD, ss715632320, was the same for all but one isolate in one generation (OH-Windfall F_8_) (**Table 6**). There are nine genes within 240 kb of this SNP marker with LRR domains. This was the largest of the four populations with each phenotypic data set containing 242 or more RILs, so it is surprising that these loci were not better defined.

**Figure 4:**
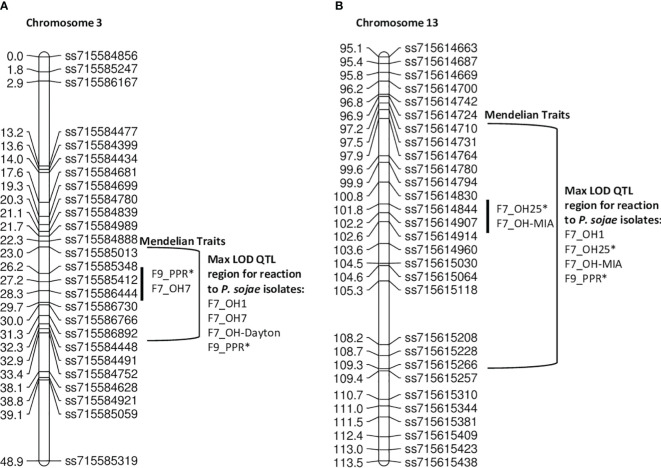
**(A)** Chromosome 3 regional map for Mendelian and QTL mapping in PI 424477 **×** Williams (positions of *Rps* loci in the inner bracket denoted by a black line). The max LOD region for reaction to isolates OH1, OH7, OH-Dayton, and PPR are denoted by the bracketed region to the right side of the map. Those isolates with an * indicates where the final results from two replications of an isolate were combined into a single dataset. **(B)** Chromosome 13 regional map for Mendelian and QTL mapping in PI 424477 **×** Williams (positions of *Rps* loci in the inner bracket denoted by a black line). The max LOD region for reaction to isolates OH1, OH25, OH-MIA, and PPR are denoted by the bracketed regions to the right side of the map. Isolates designated with an asterisk (*) indicate that disease screening results from two replications were combined into a single dataset; the remaining are from one replication.

In the **PI424477 × Williams** population, 163 RILs were phenotyped individually with five *P. sojae* isolates separately in the F_7_ generation and with the combined inoculum, PPR, in the F_9_ generation (**Table 7**, **Figures 4A, B**). Based on chi-square analysis, the resistance response to isolates OH-MIA, OH7, OH25, and PPR segregated as single *Rps* genes, while OH1 segregated as two. QTL for resistance to OH1 mapped on chromosomes 3 and 13.

**Table 7:** Resistance in PI 424477 **×** Williams toward *Phytophthora sojae* in F_7_ and F_9_ of the RIL population based on QTL mapping.

Gen. (year assayed)	*P. sojae* isolate	QTL on chr.	LOD	% explained variation	Marker at Max LOD	Flanking markers
F_7_ (2017)	OH1	3	19.46	45.7%	ss715585348	ss715585013 ss715586892
F_7_ (2017)	OH-Dayton	3	10.37	27.2%	ss715586444	
F_7_ (2017)	OH7	3	31.51	63.2%	ss715586444	
F_9_ (2023)	PPR*^+^	3	30.4	60.1%	ss715586444	
F_7_ (2017)	OH1	13	8.24	22.8%	ss715614914	ss715614710 ss715615266
F_7_ (2017)	OH-MIA	13	17.63	43.0%	ss715615030	
F_7_ (2017)	OH25*	13	24.60	52.6%	ss715614914	
F_9_ (2023)	PPR^*+^	13	7.9	21.2	ss715615030	

*Indicates where the final results from two replications of an isolate were combined into a single dataset.

^+^PPR is the combined isolates of PT2004 C2.S1 (pathotypes 1a, 1b, 1c, 1d, 1k, 2, 3c, 4, 6, 7), P7074 (pathotypes 1b, 1d, 2, 3a, 3b, 3c, 4, 5, 6, 7), and R26 (pathotypes 1b, 1d, 2, 3a, 3b, 4, 5, 6, 7, 8).

The *P. sojae* isolates OH7 and PPR mapped within 1 cM between ss715585412 and ss715586444 on chromosome 3 based on Mendelian mapping (**Supplementary Table 1**). More importantly, the marker at the max LOD score with QTL mapping for *P. sojae* isolates OH7, OH-Dayton, and PPR was ss715586444 on chromosome 3 (**Table 7**). For these three isolates, LOD scores ranged from 10.37 to 31.51, and QTL mapping explained between 27.2% and 63.2% of the phenotypic variation (**Table 7**; **Supplementary Figure 4A**). There are 28 genes annotated with leucine-rich repeats and disease resistance within this region based on Wm82.a2 sequence. Thus, there could potentially be one to two novel *Rps* genes in this region between SNP markers ss715585412 and ss715586444 that covers 1.3 Mb. Both OH-Dayton and PPR have virulence to known *Rps* genes, *Rps1a*, *Rps1b*, *Rps1c*, *Rps1d*, *Rps1k*, and *Rps7*, thus indicating a high probability of a new allele at this locus.

With Mendelian mapping, *P. sojae* isolates OH25 and OH-MIA were 1 cM apart on chromosome 13 (**Figure 4B**). The isolates OH25 and OH-MIA mapped between ss715614844 and ss715614907 (**Supplementary Table 1**). This potentially indicates that two different genes that confer resistance are present. This is supported through QTL mapping of OH1, OH-MIA, OH25, and PPR where two different markers have the maximum LOD score, ss715614914 (OH1 and OH25) and ss715615030 (OH-MIA and PPR) on chromosome 13 (**Table 7**; **Supplementary Figure 4B**). The distance between ss715614830 and ss715615030 on W82.a2 sequence assembly is 1.13 Mb and is known to be R-gene rich. There are at least 17 genes annotated with leucine-rich repeats in the W82.a2 assembly, and both OH-MIA and PPR have virulence toward *Rps3a* and *8* and PPR toward *Rps3b* and *3c*. Thus, the allele closest to ss715615030 based on QTL mapping would have the highest probability of being novel.

As noted throughout these results, we used a combination of Mendelian and QTL mapping to identify the key regions in the soybean genome for these *Rps* genes. For these advanced populations, the expected proportions of heterozygosity for a single gene should range from 0.78 to 0.19 for the F_7_ and F_9_ RIL populations, respectively. However, this did not change for the phenotypic screening and, in some cases (isolate by population), remained quite high (**Tables 2**, **3**).

Mapping by Mendelian or QTL in all four populations detected at least two resistance loci to regions covering 1 to 2 cM or 1 to 1.4 Mb on chromosomes 3, 13, and/or 18. The combined inoculum (PPR), which has virulence to all known *Rps* genes, was mapped to one or more locations in each population. Except for the chromosome 13 locus in population PI 407985 × Williams, mapping of resistance toward PPR was confirmed by another isolate mapped to the same region.

None of these loci conferred resistance to all isolates used in this study. For example, while PPR mapped toward one or more loci, other isolates had resistance segregating as single genes and mapping to alternative chromosomes. This strongly suggests that there are additional undescribed virulences or *Avr* effectors in these isolates toward more *Rps* genes than what are currently described and that differentials are available for.

## 4 Discussion

There is an essential need for continuing to identify and combine effective *Rps* genes, which have been highlighted by the fact that the widely used *Rps* genes, *Rps1a*, *Rps1c*, and *Rps1k*, are no longer effective management tools in the United States, Canada, and Argentina since the diversity of pathotypes has changed significantly over time in these areas (Dorrance et al., 2016; Grijalba et al., 2020a, b; Matthiesen et al., 2021; Tremblay et al., 2021; Hebb et al., 2022; McCoy et al., 2022, 2023). We were able to map resistance toward one and two genes toward five to seven *P. sojae* isolates in each of the RIL populations derived from four plant introductions (PI 407985, PI 408029, PI 408097, and PI 424477) previously identified as putative novel sources of disease resistance for *P. sojae* (Dorrance and Schmitthenner, 2000; Gordon et al., 2007a, 2007b; Matthiesen et al., 2016). Additionally, PI 408097 and PI 408029 are also sources of disease resistance for *Pythium sylvaticum* and *Py. irregulare* (Clevinger et al., 2021). PI 408097 is also a source of resistance to *Py. torulosum* (Clevinger et al., 2021).

Due to the number of genes segregating and the maintenance of heterozygous class in these RIL populations, both Mendelian and quantitative trait mapping were used across several generations. The mapping was similar for the LOD range and peak SNP across generations and in some populations across isolates. This phenomenon has been reported previously in studies of crosses with PIs and Williams (Gordon et al., 2007a; Ortega and Dorrance, 2011). This could be in part due to meiotic incompatibility (Ortega and Dorrance, 2011) or for other chromosomal challenges in the complex R gene loci.

Resistance toward *P. sojae* mapped in each of the four RIL populations to chromosome 3. Populations PI 407985 and PI 424477 mapped resistance qualitatively and quantitatively on chromosome 3, while in populations PI 408029 and PI 408097, resistance was mapped quantitatively. Soybean chromosome 3 continues to be the most valuable carrier of *Rps* genes against numerous isolates of *P. sojae* as resistance in this study as well as others. The SSR marker Satt009 has been reported to be closely linked to previously reported *Rps* loci on chromosome 3 including *Rps1a*, *Rps7*, *Rps14*, and *Rps1?* (Weng et al., 2001; Sugimoto et al., 2012; Lin et al., 2013; Chen et al., 2021). An SNP marker, ss715585782, in our study, is separated by 2,506 bp from Satt009 on the physical map of chromosome 3. Thus, resistance mapped to genomic regions in this study either partially or mostly overlapped where a number of *Rps* loci were previously identified including *Rps1*, *Rps7*, *Rps9*, *Rps14*, *RpsHC18*, *RpsHN*, *RpsQ*, *RpsUN1*, *RpsYD29*, *RpsYU25*, *RpsWY*, *RpsX*, and *Rps* of cv. Waseshiroge and *Rps* of cv. Daewon, and *Rps* identified in PI 407974B (Bolaños-Carriel et al., 2022; Lin et al., 2022).

A relatively large *Rps* gene containing interval on chromosome 3 was detected in the PI 424477 population, which starts at 2,921,152 and ends at 4,642,708 bp. This interval completely covers the one mapped in the PI 408097 and PI 408029 populations and mostly overlaps the *Rps* region detected in the PI 407985 population (**Supplementary Table 3**). *Rps9*, *RpsQ*, *RpsX*, and *RpsWY* loci can be added to those already mentioned above regarding commonalities between *Rps* loci map locations reported earlier and those identified in this study. Jang et al. (2020), through linkage analysis, located an *Rps* locus to a 573-kb region from 3,893,390 to 4,466,635 bp on chromosome 3 in the Daepung × Daewon RIL population. The *Rps* resistance-related sequences identified in the current study encompass this interval, which contains 10 leucine-rich repeat- and four serine/threonine protein kinase-coding genes based on Glyma.W82.a2.v1 (Jang et al., 2020).

Resistance toward *P. sojae* mapped to the *Rps3* region of chromosome 13 in all four populations. In populations PI 407985 and PI 408097, resistance loci were detected by QTL mapping for the combined inoculum, PPR, on chromosome 13. In population PI 408029, resistance loci were detected on chromosome 13 by QTL mapping for isolates OH4 and OH25. Population PI 424477 detected resistance toward isolates OH-MIA and OH25 on chromosome 13 using both Mendelian and QTL mapping. According to earlier published reports, the *Rps3* region was mapped between Satt334 (29,609,521 bp) and Satt510 (32,196,800 bp) in one study (Demirbas et al., 2001) and slightly narrowed down to Sct_033 (30,739,608 bp) to Sat_317 (32,196,800) in another report (Sugimoto et al., 2012). In this study, all isolates affiliated with chromosome 13 were mapped in the 28,859,734- to 32,225,680-bp interval as QTL and in the 29,630,754- to 30,465,386-bp interval as a Mendelian trait. The maximum LOD region on chromosome 13 for these populations is enriched with disease-related genes based on the Williams82 version sequence. Similar to the results for chromosome 3, we do expect that these loci are new alleles to these genes based on the PPR inoculum and OH-MIA as they have virulence toward *Rps3a*, *3b*, *3c*, and *8*.

The region 28,859,734–32,225,680 bp on chromosome 13 contains 33 NBS-LRR-type genes (soybase.org) and has been well studied at the sequence level (Innes et al., 2008; Ashfield et al., 2012). Resistance to several soybean pathogens is located in this region and includes *Rsv1* (Hayes et al., 2004), *Rpg1* (Ashfield et al., 1998), *Rpv1* (Gore et al., 2002), and *Rps3* (Diers et al., 1992). QTLs conferring resistance to root-knot nematodes (Tamulonis et al., 1997a, 1997b), *Sclerotinia* stem rot (Arahana et al., 2001) and corn ear worm (Rector et al., 1999), also map near this cluster of *R* genes on chromosome 13.

These locations on chromosomes 3 and 13 were somewhat unexpected in part due to the complexity and diversity of the pathotypes of the isolates used in these studies and a previous genome-wide association analysis (Van et al., 2020). Based on this analysis, 75 novel *Rps* loci were reported on all soybean chromosomes except 3 and 13 (Van et al., 2020). Chromosomes 3 and 13 were not identified in this GWAS study because many of the isolates showed virulence to *Rps* loci on chromosomes 3 and 13. In the current study, the combined inoculum, PPR, is virulent on most known *Rps* genes (vir 1a, 1b, 1c, 1d, 1k, 2, 3a, 3b, 3c, 4, 5, 6, 7, 8) and detected resistance factors on both chromosomes 3 and 13 in two of the populations, PI 407985 and PI 408097, via QTL mapping. Resistance to the combined inoculum, PPR, was on chromosome 3 in the PI 407985 and PI 424477 populations utilizing both Mendelian and QTL mapping. It is important to note that while all of the PIs were resistant to all of the individual isolates and the combination of isolates (PPR), based on the reaction of the RILs, the individual resistance genes identified in these populations, would not be effective alone toward all of the isolates. This emphasizes the need to consider stacking of *Rps* genes moving forward in cultivar development.

In two populations, PI 407985 and PI 408097, resistance to *P. sojae* was mapped to the lower arm of chromosome 18, in the same region as known *Rps* genes: *Rps4* and *Rps6* (Demirbas et al., 2001; Sandhu et al., 2004; Sugimoto et al., 2012; Zhang et al., 2013). Earlier-mapped *Rps* genes also associated with these same regions are *Rps12* and *RpsJS* (Sun et al., 2014; Sahoo et al., 2017, 2021). *Rps4* and *Rps6* are thought to be allelic, and *Rps4* co-segregates with Sat_064 (**Figure 3C** of this study: Figure 2 of Sandhu et al., 2004). In a later study, *Rps13* was also shown to be allelic to *Rps4* and *Rps6* (Sahoo et al., 2021). The *Rps* loci identified in the current study could be allelic to these *Rps* loci in the PI 408097 population as well since they are located in the same region on chromosome 18. *Rps* loci for multiple generations of OH-Windfall and one generation of OH-MIA were mapped past the known end of the long arm of chromosome 18 in both the PI 407985 and PI 408097 populations. Resistance toward isolates OH4 and OH25 also mapped to the end of chromosome 18 in the Williams × PI 407974B RIL population by Bolaños-Carriel et al. (2022).

## 5 Conclusions

Novel *Rps* genes are required to continue to use this form of resistance in areas where *Phytophthora* root and stem rot can be yield limiting. In this study, four RIL populations derived from sources of putative novel resistance with at least 163 RILs and the largest having over 240 RILs were screened for disease reaction and subsequent mapping of *Rps* genes following inoculation with *P. sojae*. Multiple single isolates of *P. sojae* were used in screening over multiple generations in three of the four populations, and a combined inoculum of three isolates (PPR) was also used to screen in these populations. PI 408029 × Williams was screened in one generation with five single isolates. The similar results observed using both Mendelian and QTL mapping and the consistency of the identified resistance-related chromosomal regions over multiple generations of each population strengthen the mapping conclusions. Based on the map location and virulence pathotype of the isolates, three novel loci were identified on chromosome 3 (PI 407985, PI 408097, and PI 424477) and two on chromosome 13 (PI 408097 and PI 424477). As the known *Rps* genes, *Rps4* and *Rps6*, on chromosome 18, could confer resistance to the isolates that mapped to this region, it is unlikely that we identified a novel gene, but an additional source of this resistance. The regions on chromosome 3 identified in three of the PIs (PI 407985, PI 408097, and PI 424477) are very important as these have multiple *Rps* genes in these regions that are closely linked together.

Several of these are putative new alleles in these regions since the populations were screened with isolates that have known virulence to these *Rps* loci in the differentials. They could be the same alleles with the more recently published *Rps* genes, but we do not have access to those genotypes currently. Sequencing is needed to verify if these results are truly the same or new alleles to some of the newly reported *Rps* genes.

As the combined inoculum had known virulence to all known *Rps* genes, this study suggests that development of new cultivars with *Rps*-mediated resistance would require the incorporation of all resistance genes from a PI.

## Data availability statement

The raw data supporting the conclusions of this article will be made available by the authors, without undue reservation.

## Author contributions

EC: Data curation, Formal analysis, Writing – original draft. RB: Data curation, Formal analysis, Investigation, Methodology, Visualization, Writing – review & editing. CS: Data curation, Investigation, Writing – review & editing. QS: Data curation, Writing – review & editing. AB: Data curation, Formal analysis, Writing – review & editing. CB-C- Data curation, Formal analysis, Writing – review & editing. AR: Methodology, Supervision, Writing – review & editing. AD: Conceptualization, Resources, Supervision, Writing – review & editing. MS: Conceptualization, Resources, Supervision, Writing – review & editing.
