# Supplementary material for: Comparison of Rps loci toward isolates, singly and combined inocula, of Phytophthora sojae in soybean PI 407985, PI 408029, PI 408097, and PI424477
Source: Front Plant Sci. 2024 Jul 1;15:1394676. doi: 10.3389/fpls.2024.1394676 (PMC11246922; doi:10.3389/fpls.2024.1394676)
Supplement: Supplementary file 1 [file Image_1.pdf]

Supplementary Figure 1. Quantitative mapping of resistance towards *Phytophthora sojae* in PI 407985 × Williams. LOD curves for A) Chromosome 3 for isolates OH1, OH12168, OH-Windfall, OH7/8 and PPR. Isolates OH1, OH12168, and OH-Windfall were assayed over multiple generations. **B)** Chromosome 18 for isolates OH1, OH25, OH-Windfall assayed over multiple generations. The year and generation assayed are designated with the isolate. The dotted line indicates the genome wide LOD threshold. Isolates designated with an asterisk (*) indicate that disease screening results from two replications were combined into a single dataset while others were from one replication.

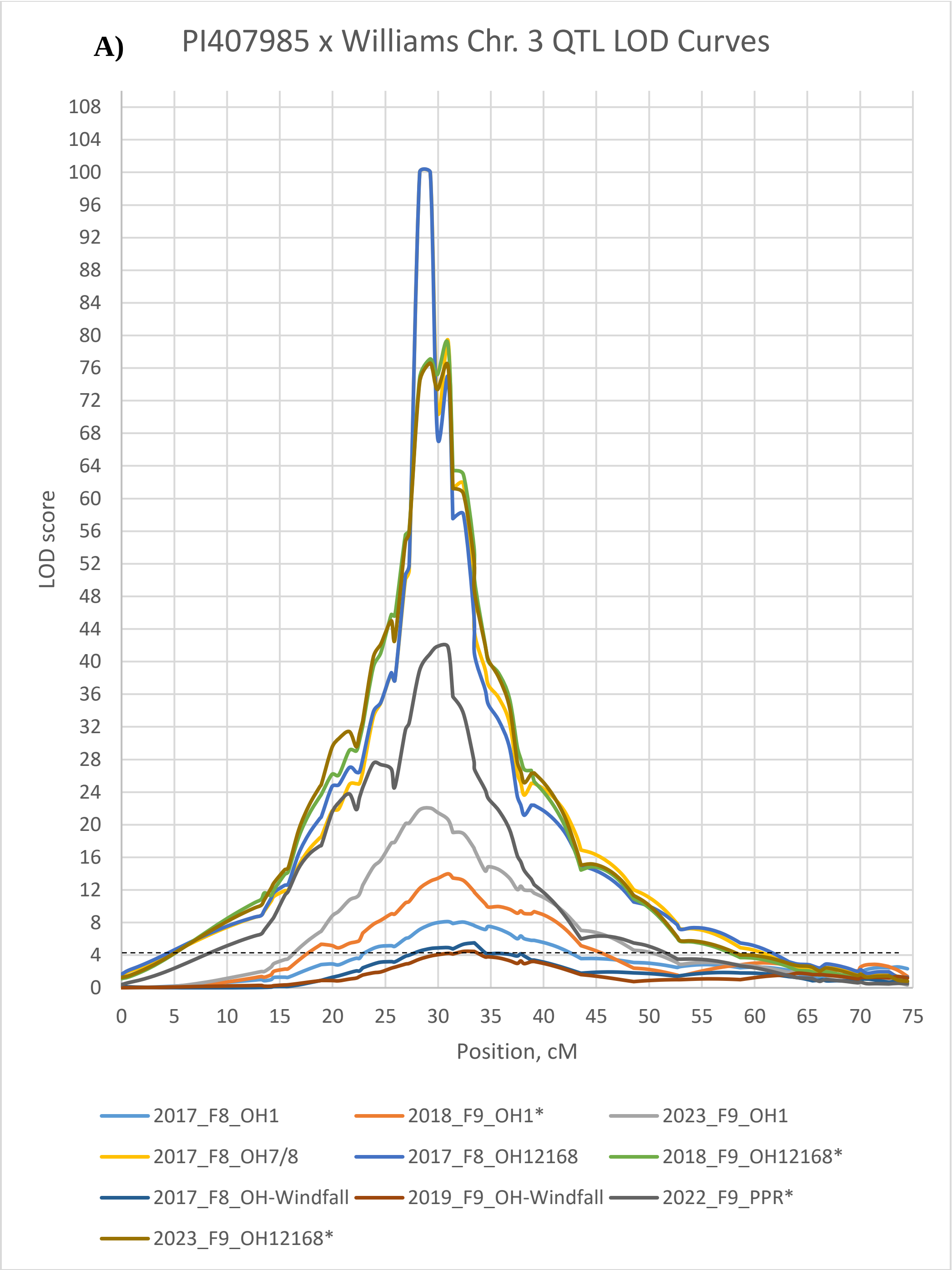

A)
PI407985 x Williams Chr. 3 QTL LOD Curves
LOD score
Position, cM
0
4
8
12
16
20
24
28
32
36
40
44
48
52
56
60
64
68
72
76
80
84
88
92
96
100
104
108
0 5 10 15 20 25 30 35 40 45 50 55 60 65 70 75
2017_F8_OH1
2018_F9_OH1*
2023_F9_OH1
2017_F8_OH7/8
2017_F8_OH12168
2018_F9_OH12168*
2017_F8_OH-Windfall
2019_F9_OH-Windfall
2022_F9_PPR*
2023_F9_OH12168*

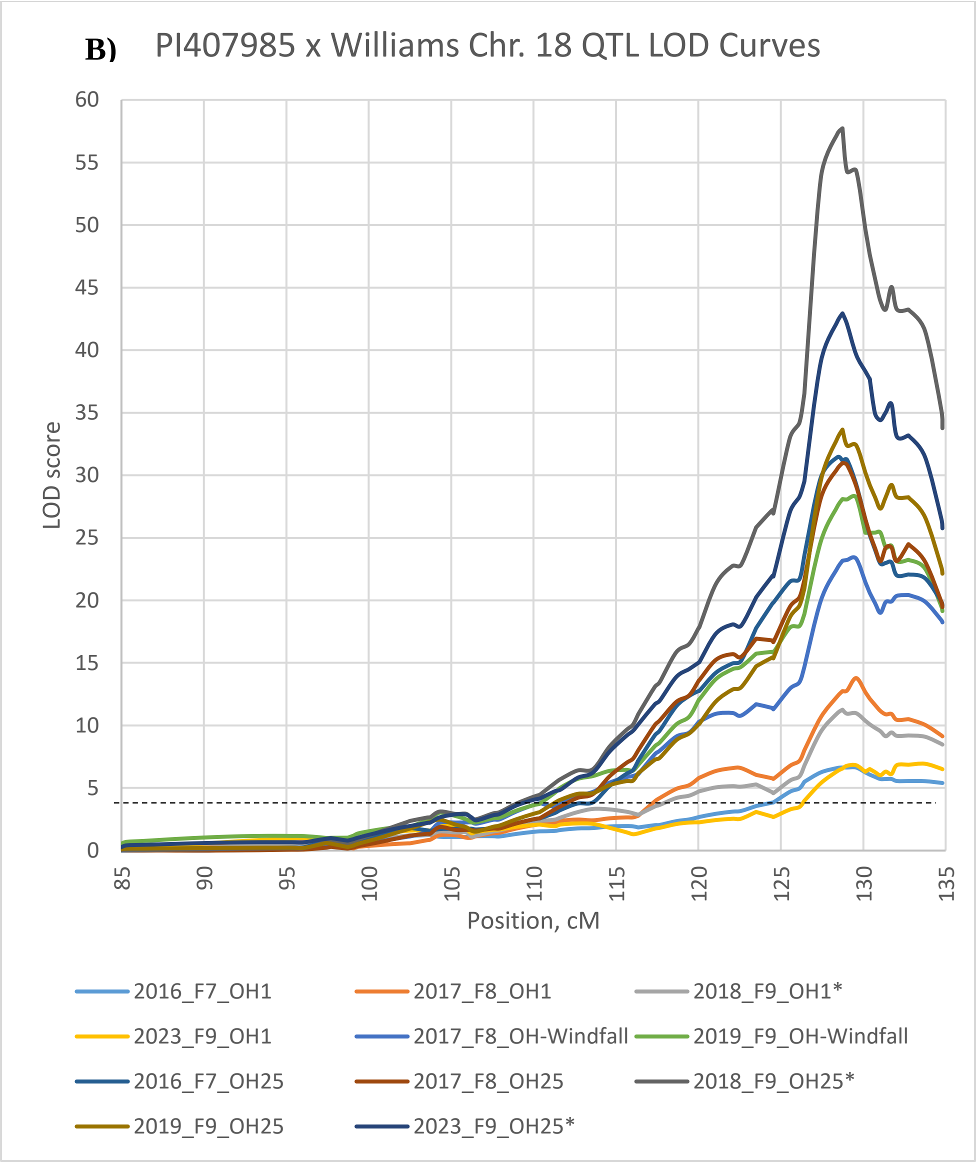
B)
PI407985 x Williams Chr. 18 QTL LOD Curves
LOD score
Position, cM
0
5
10
15
20
25
30
35
40
45
50
55
60
85
90
95
100
105
110
115
120
125
130
135
2016_F7_OH1
2017_F8_OH1
2018_F9_OH1*
2023_F9_OH1
2017_F8_OH-Windfall
2019_F9_OH-Windfall
2016_F7_OH25
2017_F8_OH25
2018_F9_OH25*
2019_F9_OH25
2023_F9_OH25*

Supplementary Figure 2. Quantitative mapping of resistance towards *Phytophthora sojae* in PI 408029 × Williams. LOD curves for **A)** Chromosome 3 for isolates OH1, OH25, OH-Windfall. **B)** Chromosome 13 for isolates OH1, OH4 and OH25. All were assayed in the F_9 generation in the population. The dotted line indicates the genome wide LOD threshold.

A)

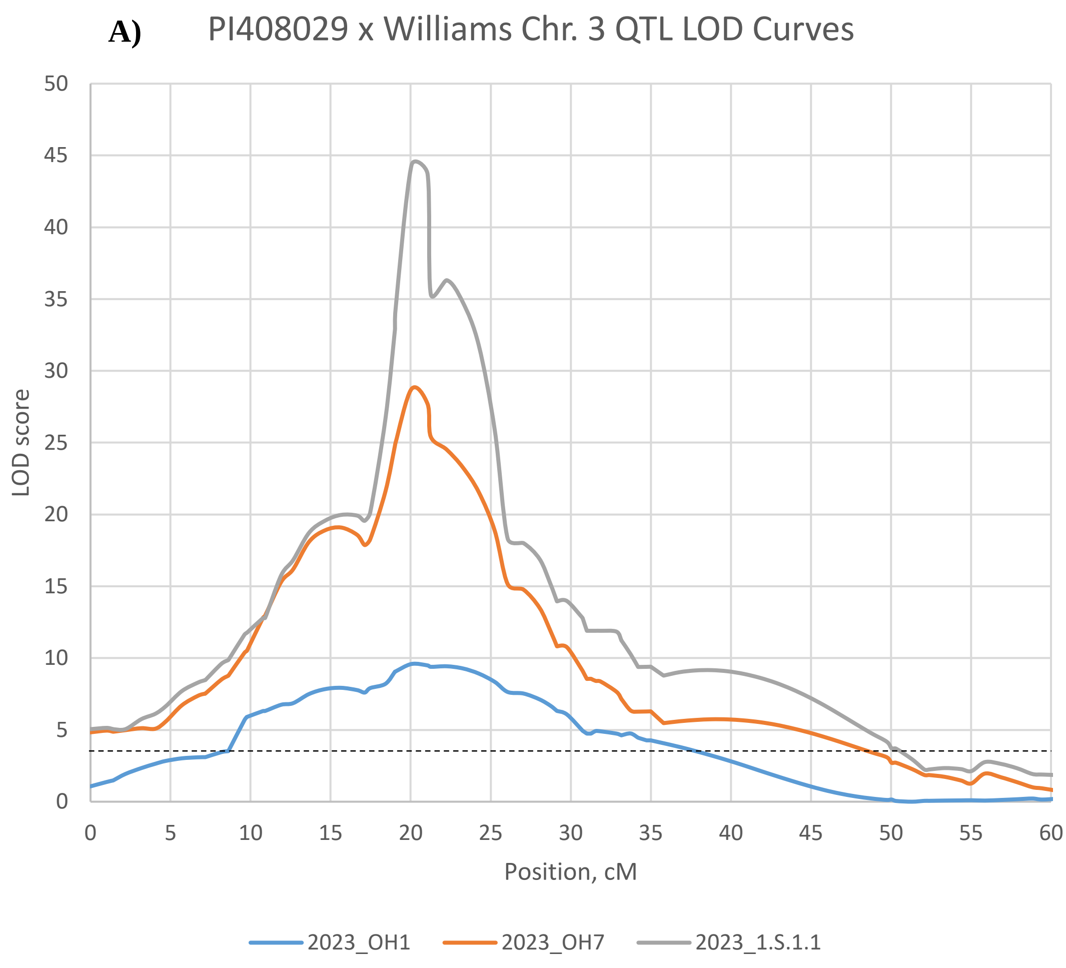

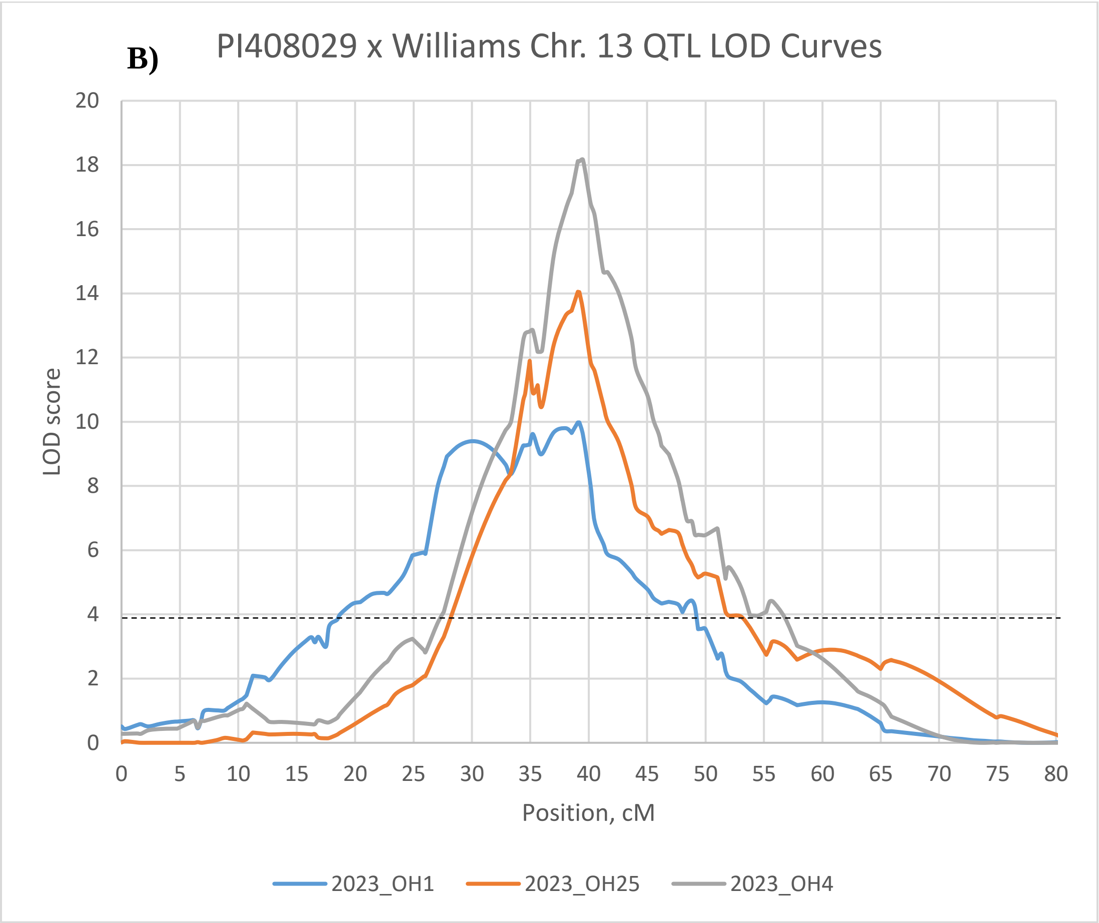
B)
PI408029 x Williams Chr. 13 QTL LOD Curves
LOD score
Position, cM
0
2
4
6
8
10
12
14
16
18
20
0
5
10
15
20
25
30
35
40
45
50
55
60
65
70
75
80
2023_OH1
2023_OH25
2023_OH4

Supplementary Figure 3. Quantitative mapping of resistance towards *Phytophthora sojae* in PI 408097 × Williams. LOD curves for **A)** Chromosome 3 for isolates: OH1, OH2, OH7, OH-Windfall, and PPR. **B)** Chromosome 18 for isolates OH1, OH2, OH-MIA, OH02017, OH-Windfall and OH-Dayton. The year and generation assayed are designated with the isolate(s) used for inoculation. The dotted line indicates the genome wide LOD threshold. Isolates designated with an asterisk (*) indicate that disease screening results from two replications were combined into a single dataset while others were from one replication.

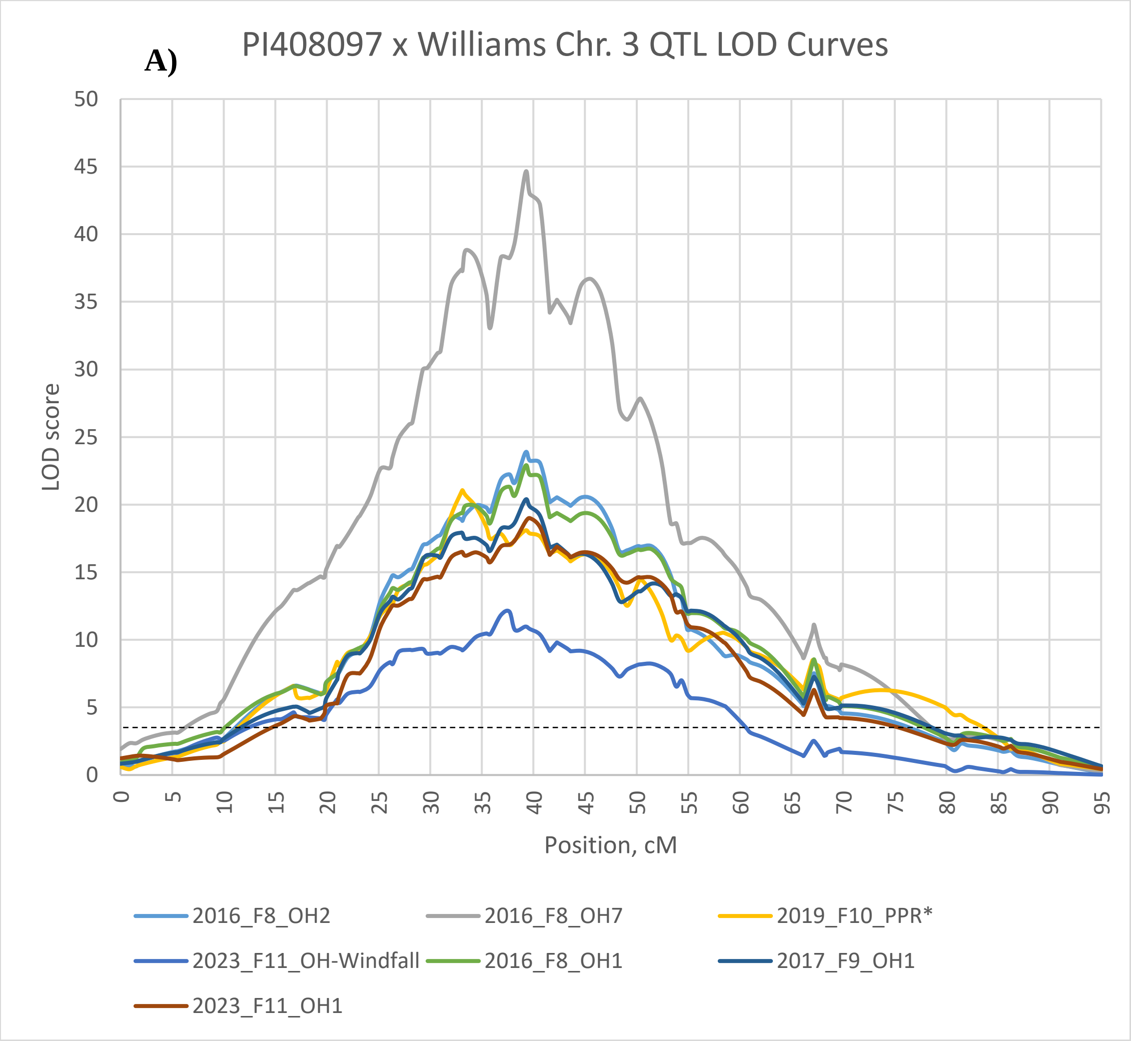

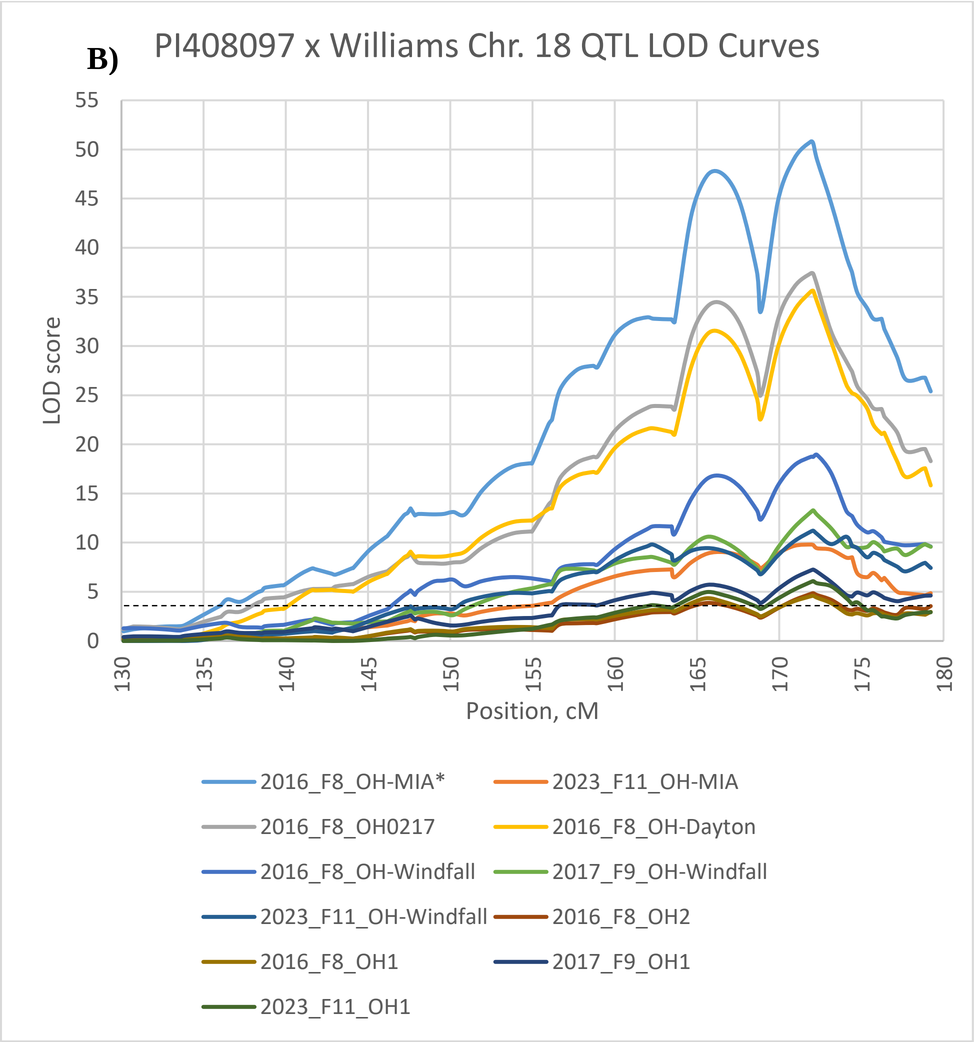

B)
PI408097 x Williams Chr. 18 QTL LOD Curves
LOD score
55
50
45
40
35
30
25
20
15
10
5
0
130
135
140
145
150
155
160
165
170
175
180
Position, cM
2016_F8_OH-MIA*
2023_F11_OH-MIA
2016_F8_OH0217
2016_F8_OH-Dayton
2016_F8_OH-Windfall
2017_F9_OH-Windfall
2023_F11_OH-Windfall
2016_F8_OH2
2016_F8_OH1
2017_F9_OH1
2023_F11_OH1

Supplementary Figure 4. Quantitative mapping of resistance towards *Phytophthora sojae* in PI 424477 × Williams. LOD curves for **A)** Chromosome 3 for isolates OH1, OH7, OH-Dayton and PPR. **B)** Chromosome 13 for isolates OH1, OH25, OH-MIA and PPR. Isolates were screened in generation F_7 except for PPR which was screened using the F_9 generation seed. The dotted line indicates the genome wide LOD threshold. Isolates designated with an asterisk (*) indicate that disease screening results from two replications were combined into a single dataset while others were from one replication.

A)

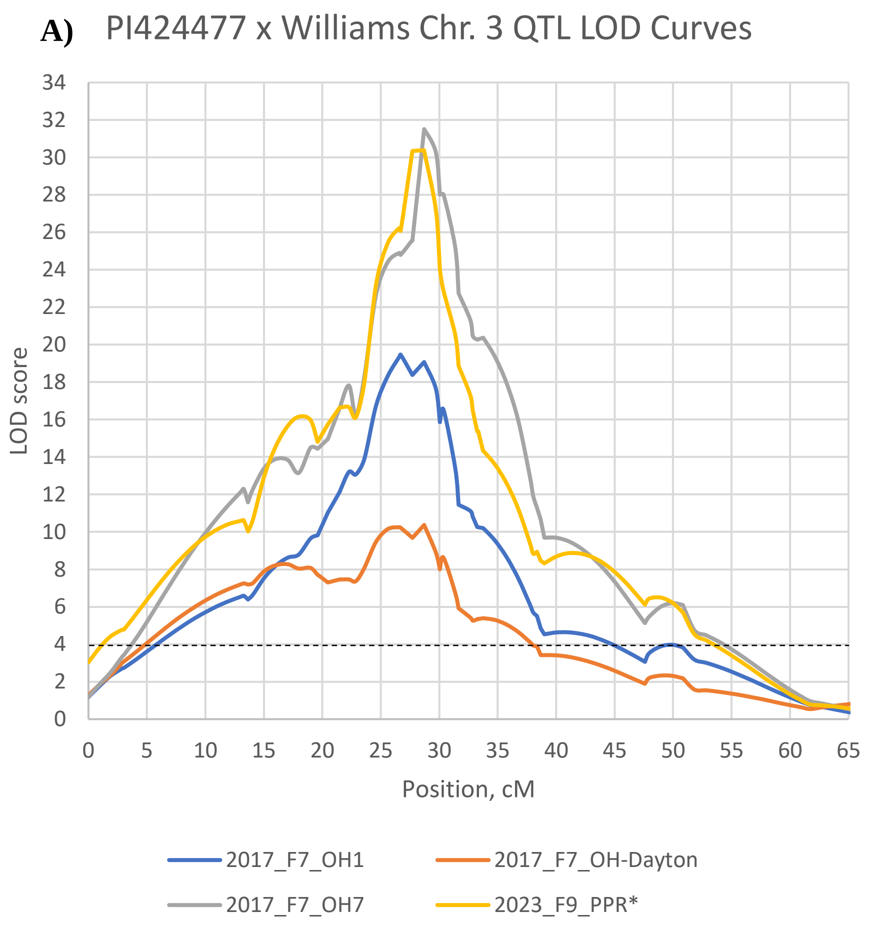

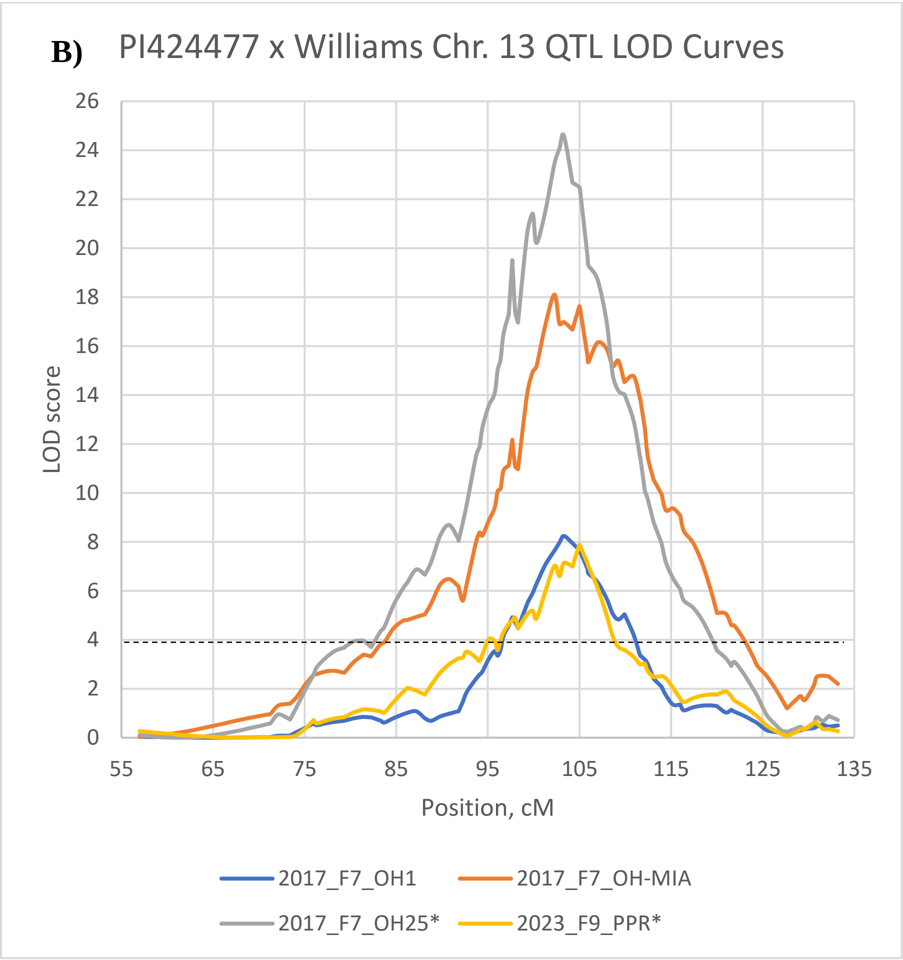
B)
PI424477 x Williams Chr. 13 QTL LOD Curves
LOD score
26
24
22
20
18
16
14
12
10
8
6
4
2
0
55
65
75
85
95
105
115
125
135
Position, cM
2017_F7_OH1
2017_F7_OH-MIA
2017_F7_OH25*
2023_F9_PPR*
