# Supplementary material for: Comparison of Rps loci toward isolates, singly and combined inocula, of Phytophthora sojae in soybean PI 407985, PI 408029, PI 408097, and PI424477
Source: Front Plant Sci. 2024 Jul 1;15:1394676. doi: 10.3389/fpls.2024.1394676 (PMC11246922; doi:10.3389/fpls.2024.1394676)
Supplement: Supplementary file 4 [file Table_3.docx]

**Supplementary Table 3.** Summary of the flanking markers and their physical positions of Mendelian and quantitative mapping of *Rps* gene-mediated resistance towards *Phytophthora sojae* isolates in four populations. All physical positions were taken from the Williams 82 *Glyma* 2.0 assembly (soybase.org).

| **Chr.** | **Population** | **Isolate** | ***Rps* as Mendelian trait**  **flanking marker IDs**  **and their physical position, bp** | ***Rps* as Quantitative trait**  **flanking marker IDs**  **and their physical position, bp** | **Maximum LOD marker of the isolate(s) within the QTL flanking marker region (physical position,bp)** |
| --- | --- | --- | --- | --- | --- |
| 3 | PI 407985 × Williams | OH12168, OH7/8, PPR | ss715585782 -ss715586806  (3,929,449 - 5,244,122) | ss715584788 - ss715584793  (2,234,147 - 16,621,811) | ss715585782  (3,929,449) |
|  |  | OH1 | - | ss715584788 - ss715584793  (2,234,147 - 16,621,811) | ss715585782  (3,929,449) |
|  |  | OH-Windfall | - | ss715584788 - ss715584793  (2,234,147 - 16,621,811) | ss715586806  (5,244,122) |
|  | PI 408029 × Williams | OH1, OH7, 1.S.1.1 | - | ss715585029 – ss715586892  (2,959,509 – 5,652,619) | ss715585348  (3,582,559) |
|  | PI 408097 × Williams | OH1, OH2, OH7 | - | ss715585102 - ss715586837  (3,115,149 - 5,354,087) | ss715586444  (4,387,476) |
|  |  | OH-Windfall | - | ss715585102 - ss715586837  (3,115,149 - 5,354,087) | ss715585412  (3,639,127) |
|  |  | PPR | - | ss715585102 - ss715586837  (3,115,149 - 5,354,087) | Satt159  (3,197,845-3,198,129) |
|  | PI 424477 × Williams | OH1 | - | ss715585013 - ss715586892  (2,921,152 - 5,652,619) | ss715585348  (3,582,559) |
|  |  | OH-Dayton | - | ss715585013 - ss715586892  (2,921,152 - 5,652,619) | ss715586444  (4,387,476) |
|  |  | PPR, OH7 | ss715585412 – ss715586444  (3,639,127 – 4,387,476) | ss715585013 - ss715586892  (2,921,152 - 5,652,619) | ss715586444  (4,387,476) |
| 13 | PI 407985 × Williams | PPR | - | ss715614755 - ss715615534  (29,128,801 - 34,518,617) | ss715615453  (33,815,541) |
|  | PI 408029 × Williams | OH1, OH25 | - | ss715614770 – ss715615208 (29,234,402 – 31,833,050) | ss715615002  (30,618,405) |
|  |  | OH4 | - | ss715614770 – ss715615208 (29,234,402 – 31,833,050) | ss715615024  (30,724,301) |
|  | PI 408097 × Williams | OH0217 | - | ss715614907 - Sat_317  (30,026,216 - 32,196,800) | ss715615118  (31,259,181) |
|  |  | PPR | - | ss715614907 - Sat_317  (30,026,216 - 32,196,800) | ss715615002  (30,618,405) |
|  | PI 424477 × Williams | OH1 | - | ss715614710 - ss715615266  (28,859,734 - 32,225,680 | ss715614914  (30,117,998) |
|  |  | PPR | - | ss715614710 - ss715615266  (28,859,734 - 32,225,680 | ss715615030  (30,765,585) |
|  |  | OH-MIA | ss715614844 - ss715614907  (29,741,893 - 30,026,216) | ss715614710 - ss715615266  (28,859,734 - 32,225,680 | ss715615030  (30,765,585) |
|  |  | OH25 | ss715614844 - ss715614907  (29,741,893 - 30,026,216) | ss715614710 - ss715615266  (28,859,734 - 32,225,680 | ss715614914  (30,117,998) |
| 18 | PI 407985 × Williams | OH1 | - | ss715632295 – BARCSOYSSR_18_1949  (56,127,446 – 57,972,957) | ss715632359  (56,570,437) |
|  |  | OH1 | - | ss715632295 – BARCSOYSSR_18_1949  (56,127,446 – 57,972,957) | ss715632312  (56,299,103) |
|  |  | OH1 | - | ss715632295 – BARCSOYSSR_18_1949  (56,127,446 – 57,972,957) | ss715632492  (57,679,910) |
|  |  | OH25 | ss715632312 – ss715632359  (56,299,103 – 56,570,437) | ss715632295 – BARCSOYSSR_18_1949  (56,127,446 – 57,972,957) | ss715632312  (56,299,103) |
|  |  | OH-Windfall | Maps Below  BARCSOYSSR_18_1949 (57,972,957) | ss715632295 – BARCSOYSSR_18_1949  (56,127,446 – 57,972,957) | ss715632359  (56,570,437) |
|  | PI 408097 × Williams | OH1,  OH-Dayton, OH2 | - | ss715632295 - BARCSOYSSR_18_1949  (56,127,446 - 57,972,957) | ss715632320  (56,346,353) |
|  |  | OH0217,  OH-MIA | ss715632312 - ss715632320 (56,299,103 - 56,346,353) | ss715632295 - BARCSOYSSR_18_1949  (56,127,446 - 57,972,957) | ss715632320  (56,346,353) |
|  |  | OH-Windfall | Maps Below  BARCSOYSSR_18_1949 (57,972,957) | ss715632295 - BARCSOYSSR_18_1949  (56,127,446 - 57,972,957) | ss715632320 & ss715632339 (56,346,353 & 56,443,425) |
